# Not all numbers are equal: preferences and biases among children and adults when generating random sequences

**DOI:** 10.3389/fpsyg.2014.00019

**Published:** 2014-01-23

**Authors:** John N. Towse, Tobias Loetscher, Peter Brugger

**Affiliations:** ^1^Centre for Research in Human Development and Learning, Department of Psychology, Lancaster UniversityLancaster, UK; ^2^School of Psychology, Flinders UniversityAdelaide, Australia; ^3^Department of Neurology, University Hospital ZurichZurich, Switzerland; ^4^Zurich Center for Integrative Human Physiology, University of ZurichZurich, Switzerland

**Keywords:** numerical cognition, spatial cognition, random generation, cognitive development, number preferences

## Abstract

We investigate the number preferences of children and adults when generating random digit sequences. Previous research has shown convincingly that adults prefer smaller numbers when randomly choosing between responses 1–6. We analyze randomization choices made by both children and adults, considering a range of experimental studies and task configurations. Children – most of whom are between 8 and 11~years – show a preference for relatively large numbers when choosing numbers 1–10. Adults show a preference for small numbers with the same response set. We report a modest association between children’s age and numerical bias. However, children also exhibit a small number bias with a smaller response set available, and they show a preference specifically for the numbers 1–3 across many datasets. We argue that number space demonstrates both continuities (numbers 1–3 have a distinct status) and change (a developmentally emerging bias toward the left side of representational space or lower numbers).

## INTRODUCTION

There is a strong and venerable interest in mathematical cognition and its development. Out of a large body of empirical work, several important and richly developed theoretical accounts of number processing exist, including for example, the triple-code model (Dehaene and Cohen, 1995) and the encoding complex approach (Campbell, 1994). Such theories make important predictions for mathematical processing in particular. Yet they rest on a sound understanding of how numerical values are represented. For example, numbers involve core mathematical properties, such that with the highly familiar example of a ruler, each value is equally distanced from others. Yet it need not be the case that in psychological space, the same metrics – or internal spaces – apply. In the present paper, we demonstrate that indeed not all numbers are equal; some values are more easily accessible than others, for children and adults. Although number preferences have previously been demonstrated for adults, we show important differences – as well as continuities – in biases exhibited across development. At the same time, we will present data that show preferences are not fixed or immutable. In particular, the “attractiveness” of numbers is not simply an inherent feature of those numerical representations. We will show that preferences need to be understood with reference to the presence or absence of numerical neighbors, as well as the researchers’ decision about how to label numerical sets.

An interesting test case for exploring the properties of number representations comes from random number generation tasks. There is a very extensive literature on the psychological interpretations of attempts to produce random choices. Reviewing the work at the time, Brugger (1997) considered almost 300 research papers, demonstrating clear effects from task difficulty, and performance changes as a function of mental health status. Much work has been conducted since that review, making a comprehensive summary impractical here. Suffice to say that different response choices have been studied, including letters of the alphabet, temporal gaps, and spatially arranged keys. These production formats affect performance (Towse, 1998; Vandierendonck, 2000; Vicario, 2012), yet number sequences form a convenient, popular and relatively well-understood choice set. This random number generation task is typically used as a measure of executive functions, stressing for example the capacity to inhibit prepotent sequences (such as neighboring values) and repetition avoidance (the reluctance to reuse numbers quickly) – see Towse and Neil (1998) for indications of the factor structure underlying performance, and Miyake et al. (2000) for a seminal model of executive functioning that incorporates data from a random generation task.

Typically, random generation research dwells on the *sequential structure* in responses, and/or the relative accessibility of the response set. Insofar as participants in the random number generation task are making repeated number selections, their response choices might serve additional functions. First, they can potentially reveal numerical preferences independently of randomization quality, and second, they may contribute to an understanding of the potential links between numbers and mental space. With respect to the first point, Loetscher and Brugger (2007) re-analyzed data from 16 experiments in which adults completed a Mental Dice Task (MDT), random generation of 66 verbal responses involving the numbers 1–6. Their analysis of the *distribution* of response choices showed a systematic bias; participants showed a preference for small numbers (i.e., responses “1,” “2,” or “3”). Moreover, this small number bias (SNB) was quantitatively present in every dataset, and was significant for six of the individual datasets. The average surplus of small numbers was 0.68, quantitatively small but highly significant overall. Di Bono and Zorzi (2013) have recently replicated the SNB with a verbal random number task involving the numbers 1–9, while Vicario (2012) has reported data on a random keypress task requiring 90 response choices among all eight fingers mapped onto eight keys. Vicario reported overall a right hand (i.e., larger value) preference, too.

Loetscher and Brugger (2007) were unable to distinguish between several potential explanations of the SNB. However, one suggestion is that a preference for smaller numbers may reflect a combination of (a) the spatial nature of number representations (left to right in going from small to large) and (b) the biologically rooted left-sided exploration bias in space (Jewell and McCourt, 2000). Several sources of evidence point to the spatial coding of internal number representations. These include the spatial numerical association of response codes (SNARC) effect (see Dehaene et al., 1993; Wood et al., 2008; Fischer and Brugger, 2011; Patro and Haman, 2012), magnitude classification tasks (Moyer and Landauer, 1967), the impact for number processing of neuropsychological damage to areas involved in spatial processing (Zorzi et al., 2002; Loetscher et al., 2010) and research on children’s linear and non-linear number line estimations (Siegler and Opfer, 2003).

With respect to the second issue – the link between number preferences in random generation and mental space – Loetscher and Brugger (2007) also established that individual SNBs were correlated with leftward deviations in number line bisection and in some participants, with orientation biases in real space. Moreover, SNB magnitude can be affected by verbal or spatial attention load, and for example left head turns result in the generation of smaller numbers (Loetscher et al., 2008; see also Takio et al., 2013; for evidence that attentional load affects spatial biases in children). Finally, research focusing on the mental number line often suggests some element of spatial compression such that smaller numbers have greater weighting (Banks and Hill, 1974; Siegler and Opfer, 2003).

Two key issues from Loetscher and Brugger (2007) motivate the current analyses. First, they explicitly identified developmental data as important, in order to investigate whether number preferences are acquired through gradual experience or not. For example Wood et al. (2008) suggested the SNARC effect is not reliably found before approximately 9.5 years of age (see also van Galen and Reitsma, 2008). We are not aware of any published data on number biases in children’s random number generation, and thus we investigate children’s task performance in the present paper.

Second, Loetscher and Brugger acknowledged that their analyses were based on the MDT in which there were always six responses. Consequently, it is not known whether or how number preferences are shaped by particular task configuration. Although we have noted the use of different response choices in other work, the impact of this variable has not been considered in any detail. For example, in the context of studying number preferences among digits 1–6, their label “small” might be used in an absolute sense (i.e., there is something special about the first three digits regardless of the range of numbers being considered) or in a relative sense (i.e., for a given range, numbers smaller than the median as opposed to numbers larger than the median). To address these questions requires data from tasks that involve different response set ranges. We consider such data here.

We investigate number preferences through secondary analysis of several children and adult datasets involving random number generation experiments. These involve a suite of experimental manipulations, including the variation of response set size. Rather than amalgamating all possible datasets straight away, we initially describe a complementary set of studies involving response choices 1–10. We subsequently describe adult performance also using this response range, and then children using responses 1–7. This sectioning permits a focus on a range of important issues.

## ANALYSIS 1: NUMBER PREFERENCES AMONG CHILDREN

We took advantage of the opportunity to (re) analyze existing random generation data from children. The most common procedure is to ask children to choose among 10 alternatives (i.e., respond with numbers 1–10) and so we begin with this format.

### MATERIALS AND METHODS

#### Participants

The present analysis comprises 225 children who verbally produced random sequences using responses between 1 and 10 (at least as part of their task session; n.b. This allowed us to include some extra children in analyses who had to be excluded from the original paper because of missing or problematic data in other conditions). These datasets are described in **Table 1**, ordered by mean sample age.

**Table 1:** Small/large number biases in children’s number preferences.

Study (*n* = sample size)	Age range	Frequency of small numbers	Frequency of large numbers	*t*-Test
Towse and Mclachlan (1999a, E3) (*n* = 33)	(4;11–7;6)	32.5	37.5	2.13*
Towse and Mclachlan (1999b) (*n* = 508)	(8;2–11;1)	34.3	35.7	1.20
Towse and Mclachlan (1999a, E1) (*n* = 42)	(8;3–11;2)	33.8	36.2	1.79+
Towse and Mclachlan (1999a, E2) (*n* = 36)	(8;4–11;3)	34.0	36.0	1.69+
Unpublished data (*n* = 64)	(11;10–16;1)	37.1	37.9	0.78
**Mean *surplus* of responses 1, 2, and 3**				***t*-Test**
Towse and Mclachlan (1999a, E3)	0.42			0.49
Towse and Mclachlan (1999b)	0.06			0.11
Towse and Mclachlan (1999a, E1)	0.10			0.17
Towse and Mclachlan (1999a, E2)	0.50			0.88
Unpublished data	0.70			1.61

The *t*-test compares the small with large numbers (upper table) and the frequency of first three numbers compared with the average of all responses (lower panel). The symbol “*”indicates *t*-test is significant at *p* < 0.05, “+”indicates *t*-test effect 0.05 < *p* < 0.10.

The youngest group was reported as Experiment 3 in Towse and Mclachlan (1999a), with datasets from Towse and Mclachlan (1999b) and Towse and Mclachlan (1999a) [originally described as Experiment 1 (the slow condition) and Experiment 2 respectively]. Finally, we analyze data from an unpublished study of secondary school children (part of an experiment manipulating response speed among children).

The age range of the composite sample varies considerably, between 4 years 11 months and 16 years 1 month (see **Table 1** for age ranges). All children produced 70 responses in a sequence except for the final-mentioned group who produced 75 responses. Responses were cued by an auditory tape comprising a systematic sequence of computer beeps. The inter response interval was 2.5 s for all datasets, except for the youngest group, where the interval was 1.5 s. Responses were monitored by the Experimenter who recorded any omissions or errors (i.e., responses outside the range), data that are not considered here.

### RESULTS

**FIGURE 1:**
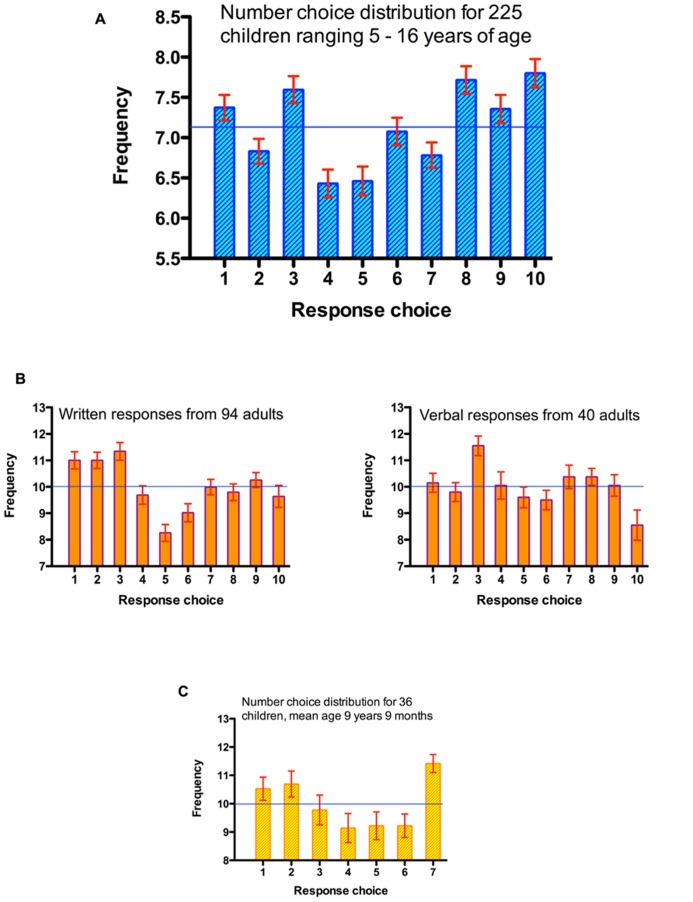
**Distribution of response choices when attempting to generate random sequences.** **(A)** Aggregated data across datasets from children when selecting among the numbers 1–10. **(B)** Adult preferences when selecting among the numbers 1–10, with written and verbal response modes. **(C)** Children’s preferences when selecting among the numbers 1–7. Horizontal lines represent chance performance.

Response preferences across the corpus are reported in **Figure 1**. There was overall, a *bias toward larger numbers* (i.e., the frequency of response choices 1–5 < 6–10). There was a mean excess of 2.03 larger numbers relative to smaller numbers, *t*(224) = 3.38, *p* = 0.001, η^2^ = 0.049. **Table 1** describes the number preference data for each study cohort separately. Every dataset produced, descriptively, a large number bias (LNB), albeit only in one case was this significant at the individual study level. Notably, that involved the youngest age group (mean age 6;5) whilst the smallest bias occurred among the oldest group (mean age 14;1). Across all these data, we found a small but reliable correlation between children’s age and the size of the SNB, *r*(223) = 0.149, *p* = 0.025.

Therefore, in contrast to results found with adult samples, children overall show a reliable LNB, not an SNB. However, previous analyses have used the MDT in which there were six response alternatives, and thus small numbers were defined as 1, 2, and 3. We therefore investigated specifically whether numbers 1–3 were used preferentially. We refer to this as a first numbers bias (FNB), potentially reflecting a preference for the earliest acquired numbers. Indeed, the first three numbers were produced more often than the average of all ten response alternatives (mean FNB = 0.373 items), but this was not a statistically reliable effect, *t*(224) = 1.51, *p* = 0.132, η^2^ = 0.010. None of the individual study analyses showed significant biases either, though all trends were in the direction of an FNB. A third, alternative analytic approach is to follow the MDT procedure literally and compare the frequency of responses 1–3 (*M* = 21.8) with 4–6 (*M* = 20.0; thereby ignoring number choices 7 through 10). This produced a strong FNB, *t*(224) = 4.32, *p* < 0.001, η^2^ = 0.075.

In sum, almost paradoxically, we find both a preference for numbers 1–3, and a preference for the larger-than-the-median numbers. Clearly this emphasizes the importance of the operational definition of “small.” It also demonstrates that the SNB reported previously is not a universal preference, once data from children are considered.

### DISCUSSION

*A priori*, we anticipated an SNB, or perhaps the developmental emergence of an SNB from a baseline of no preference. Such an outcome would be consistent with the acquisition of number space correspondences as children become increasingly familiar with numerical material. Instead, what we actually observed was that when choosing between numbers 1–10, children showed an LNB, where large is defined by a median-split with respect to the range of response options. We also found a weak but systematic developmental trend for the emergence of small number preferences. In addition we obtained evidence that number preferences vary with the way magnitude contrasts are constructed. If “small” is defined instead with reference only to the numbers 1–3, then the picture changes yet again, with more evidence for a bias toward these first, or early acquired numbers.

The current analysis has focused on response choices with the numbers 1–10 since this allows for analysis of the largest aggregated set of data. Given the apparently contrasting findings to those reported from adults by Loetscher and Brugger (2007; see also Di Bono and Zorzi, 2013), we next report corresponding random generation data from adults who also used numbers 1–10. This allows us more clearly to distinguish developmental differences from task configuration effects. We chose to analyze two datasets from Towse and Valentine (1997). One involved written responses, a further study elicited verbal sequences.

## ANALYSIS 2: NUMBER PREFERENCES AMONG ADULTS

### MATERIALS AND METHODS

#### Participants

There were 94 adults described in Experiment 1 of Towse and Valentine (1997), who generated 100 responses from numbers 1–10. Responses were written into a booklet that had space for 10 responses on each page (and thus 100 in total). In Experiment 2, 40 adults generated verbal sequences. In both studies, responses were produced with an interval of 1.5 s between each response cue (a computer beep). In the first study, an Experimenter oversaw the data collection in a group (there were more than 100 response cues to account for any occasional response lapses by participants). In the second study, an Experimenter recorded the responses and monitored compliance with response cues.

### RESULTS

Response preferences are shown in **Figure 1B**. Combining datasets, adults show a systematic preference for numbers 1–5 (*M* = 51.3) compared to 6–10 (*M* = 48.7), *t*(133) = 3.00, *p* = 0.003, η^2^ = 0.063. That is, they showed an SNB. This was true for the larger individual dataset also, *t*(93) = 2.38, *p* = 0.019, η^2^ = 0.057, while the trend in the other dataset fell short of significance, *t*(39) = 1.98, *p* = 0.055, η^2^ = 0.091, though the effect size was larger.

Analysis also confirmed what is evident by inspection of **Figure 1B**; the first three numbers were particularly popular choices. Adopting the analytic approach used in the previous analysis (comparing the use of numbers 1–3 with all others) there was an FNB with the aggregated data, *t*(133) = 7.29, *p* < 0.001, η^2^ = 0.285, and for both individual datasets [*t*(93) = 6.83, *p* < 0.001, η^2^ = 0.334, and *t*(39) = 2.74, *p* = 0.009, η^2^ = 0.185]. In all cases these FNB effect sizes are larger than the SNB effects based on a median-split contrast between small and large categories.

Third and finally, as with the children’s data, we also compared just numbers 1–3 with numbers 4–6 (i.e., using only the number available in the MDT, and thus ignoring responses 7–10). This confirmed an FNB, *t*(133) = 7.57, *p* < 0.001, η^2^ = 0.301. A systematic FNB was obtained for the individual datasets also [*t*(93) = 7.45, *p* < 0.001, η^2^ = 0.374, and *t*(39) = 2.39, *p* = 0.022, η^2^ = 0.128].

### DISCUSSION

The adult data provide an important context for the findings and potential conclusions in Analysis 1. They confirm the SNB in adults, as previously established by Loetscher and Brugger (2007) as well as by Di Bono and Zorzi (2013). The current replication is obtained from sequences involving ten response alternatives. Children’s LNB in Analysis 1 cannot merely be explained as an idiosyncrasy of the response set used (1–10), compared for example to the original choices 1–6 analyzed by Loetscher and Brugger (2007). At the same time, the data also show that, as for children’s data, it potentially matters whether one defines small in relative terms (below the median; SNB) or in absolute terms (the numbers 1–3; FNB). The bias (i.e., the effect size) was particularly large when considering the first three numbers.

These conclusions are drawn from two existing datasets of adult random generation performance. It is important to recognize the differences in response format in these cases. SNB tended to be larger for written compared to oral responses (cf. **Figure 1B**). This could be due to the fact that in the written condition, the participant could review up to ten preceding responses on the page. In the verbal response condition, adults could only utilize memory representations. Hence, in the written condition, perceptual-representational effects of pseudoneglect (Jewell and McCourt, 2000) may have added to the SNB. Prospective studies are needed to systematically investigate the potential influence of response mode on asymmetries in number space. Key to the present paper is that systematic biases were evident in both conditions.

In the final set of analyses, we draw on one specific and highly relevant dataset; where children were asked to generate a random sequence with just seven choices. This provides the closest known match for children to the MDT, and allows us to ask specifically: do children show an SNB when they choose amongst a response set similar to that reported by Loetscher and Brugger (2007) among adult participants?

## ANALYSIS 3: NUMBER PREFERENCES AMONG SMALL RESPONSE SETS

### MATERIALS AND METHODS

Data are considered from 36 children who were given seven response choices and produced 70 randomization responses (Towse and Mclachlan, 1999a, Experiment 2; note these children also contributed sequences with 10 responses in Analysis 1). Computerized beeps from a tape recording, with a 2.5 s interval, formed the response cue, which was monitored by the Experimenter who recorded the children’s verbal choices.

### RESULTS

When children had just seven response choices available, the contrast between an SNB (1–3 vs. 5–7) and FNB (1–3 vs. others) is of course less meaningful. Nonetheless, for consistently across the analyses conducted for each dataset, we describe the analytic contrasts as before (see **Figure 1C**, for response distributions). Children produced more numbers smaller than the median (*M* = 31.0), than they did numbers larger than the median (*M* = 29.9), but this SNB bias was not significant, *t*(35) = 1.29, *p* = 0.204, η^2^ = 0.046. Children chose the values 1, 2, and 3 more frequently than the average of all responses; this FNB was significantly different from 0 (mean excess of choices = 1.00), *t*(35) = 2.20, *p* = 0.019, η^2^ = 0.122. Finally, as with previous analyses, we also compared the use of numbers 1–3 with the use of 4–6 by analogy with the MDT; smaller numbers were again preferred (*M* = 31.0 vs. 27.6 choices), *t*(35) = 3.39, *p* = 0.002, η^2^ = 0.247.

### DISCUSSION

With respect to Analysis 3 specifically, we have shown that children can exhibit a SNB in their random number generation preferences. Analysis 1 reported a LNB among children, which stands in stark contrast with published adult data using the MDT (Loetscher and Brugger, 2007). The present data confirm that when children’s response set is more similar to that used in the adult studies, we find a much closer alignment of biases. Number preferences are strongest when there is a direct comparison of the MDT by using numbers 1–6 in the present data. The size effect is emphasized because responses 1–3 are popular while responses 4–6 are less popular and the analysis omits consideration of the (popular) response 7 from the large set.

Thus, the range of options available to the individual affects how preferences become scattered across the alternatives. It is perhaps worth noting that for these children specifically in Analysis 1, responding in 10-choice task, they exhibited a (marginally significant) LNB. Thus there is a within-sample contrast as well as a difference that emerges between separate datasets. This further emphasizes that the number preference biases are sensitive to the eliciting conditions, including the range of responses available on the task.

Reflecting on all the datasets and all three sets of Analysis, we argue that our investigation of number preferences within random generation sequences serves multiple purposes and illuminates a range of important issues. First, the results confirm that number preferences exist among children. Numbers are not all equally “attractive” when children attempt to create a random sequence. Second, it is apparent that those preferences are not necessarily the same for children and adults. In particular, whilst previous research has identified a reliable and systematic SNB among adults, and we confirm an SNB for adults who select from numbers 1–10, children across multiple datasets preferentially call on larger numbers (at least where “large” is defined with respect to numbers greater than the median). There is also a small but consistent developmental increase in the bias toward small numbers.

Third, we argue that in terms of number preferences there seems to be something different about the numbers 1–3, insofar as they represent particularly attractive selections in a random generation task for children and adults. When children have a small response set (the numbers 1–7), then the numbers 1–3 predominate in choices. We refer to this as an FNB. These numbers are early acquired, and as a result they may be represented most strongly (see also Di Bono and Zorzi, 2013). These findings are also consistent with the contention from Dehaene (2011) that there are multiple core number systems, including a small number system (perhaps an object tracking system) for the numbers 1, 2, and 3. This might lead to small numbers being over-accessible in production tasks including random generation.

Fourth, the present analyses replicate reports that an SNB exists among adults. This bias is more than just a preference for the numbers 1–3 (i.e., we report both SNB and FNB effects in adult data). We therefore conceptually replicate the adult pattern from the MDT originally described by Loetscher and Brugger (2007; see also Loetscher et al., 2008; Di Bono and Zorzi, 2013).

Fifth, the present analyses emphasize that number preferences can vary as a function of the task configuration, in particular in terms of the number of response alternatives available. It is *not* the case that conclusions from Loetscher and Brugger (2007) apply only to the MDT where individuals choose between digits 1 and 6. Nonetheless, as explicitly cautioned in that paper, the results there may not always generalize to different sets. Number preferences and number choices are affected by the choices available; numerical neighbors can affect the status of a representation as being more or less accessible.

We have drawn upon existing datasets in which children (and adults) have generated random sequences. This has allowed us to establish, among other things, that the previously reported SNB in adults’ sequences is not a universal preference. Children do not always show the same bias – indeed, they sometimes exhibit a systematic bias in the other direction. Also, we found a correlation between age and the strength of the SNB. This leads us to conclude that there is a gradual change across development that leads to the adult performance profile. We are not in a position to identify either the catalyst for this change in number preferences, nor exactly the period when the change occurs. Most of the children analyzed here are between 8 and 11 years of age, so we can be reasonably confident that adult-like number preferences do not emerge until late in development. Our working hypothesis is that the SNB emerges with ever-increasing familiarity with the mental number line (Wood et al., 2008), and with the accessibility of the first three numbers in particular. Our finding that adults produce both ascending and descending responses sequences, while children produce mostly ascending sequences, is testament to how number relationships are becoming more automatized.

Yet the present findings clearly invite an explanation as to why children show a reliable LNB, as opposed to finding the developmental emergence of an SNB. We suggest there are at least two components to this effect. First, for reasons that are not entirely clear, children show a reluctance to choose the numbers 4 and 5 – see **Figure 1**. This may be partly an anchoring effect, where “middle” values are avoided. Whatever the cause, the consequence is that numbers less than the median in a ten-choice task become underrepresented, and it also explains why a focus just on numbers 1–3 does not yield a FNB.

Second, in generating random sequences, we note that previously published Figures imply that there may be developmental differences in the production of ascending and descending “runs” of numbers in random sequences. Indeed, analyzing such data formally, adult data (see the “-1” and “+1” contrast in **Figure 1**, Towse and Valentine, 1997), reveal more descending neighbor sequences (e.g., “7” then “6”) than ascending sequences (e.g., “6” then “7”; *M* = 14.1 vs. 12.7 respectively) *t*(94) = 2.48, *p* = 0.015, η^2^ = 0.061. In contrast, children show the opposite pattern; a predominance of ascending sequences (see Towse and Mclachlan, 1999a, **Figure 1**). Combining fast and slow responses from Towse and Mclachlan (1999b; Experiment 1) generates *M* = 30.7 and *M* = 23.4 for ascending and descending neighbor run frequencies, *t*(42) = 2.82, *p* = 0.015, η^2^ = 0.159. This is not that surprising; descending number runs form a less overlearned response chain for children, added to which there is a developmentally emerging left-to-right preference in counting that would be compatible with a small-to-large number choice (see Shaki et al., 2012). We suggest this might yield a “drift” of number selections toward the larger boundary since after an ascending run the selection of subsequent choices do not always revert to the very lowest number. At the same time this cannot be a complete explanation of preferences for large numbers however. Children show an SNB with a 7-item response set in Analysis 3, which is not accommodated by this account.

As noted by Loetscher and Brugger (2007) a retrospective/secondary analysis of number preferences has both advantages as well as limitations. One major advantage is that experimental demand effects are unlikely to contribute in a major way to findings. However, one limitation is that children were asked *only* to generate random sequences. As a consequence, ancillary data is not available in which other assessments of representations of space such as line bisection can be used to derive estimates of “pseudoneglect” (e.g., Jewell and McCourt, 2000; van Vugt et al., 2000). In future, additional tasks are likely to form an important complement to number choice data. For example, on the basis of physical and mental bisection tasks, Göksun et al. (2013) have suggested that there may be separate spatial attentional mechanisms among children that become integrated across development. Accordingly, number preference data might help test such an account.

Thus, we believe the current research opens up the potential for various new and exciting opportunities for understanding number preferences. It does so, we argue, on the foundational insights developed here. We have shown for the first time with random generation data that, among children, not all number are equal. Yet the preferences for children and adults are not necessarily the same, and there is a developmental transition in response profiles. In addition, the available response set makes a potentially critical difference to the preferences that are exhibited, as does the way in which the “small” and “large” contrast is developed methodologically. In all these novel findings, the analyses illustrate how the present research can help both to constrain theoretical accounts of number preferences, and inform how mental representations of number are linked to broader theories of magnitude (Walsh, 2003).

## Conflict of Interest Statement

The authors declare that the research was conducted in the absence of any commercial or financial relationships that could be construed as a potential conflict of interest.
